# Serum Sestrin2 Was Lower in Septic Shock Patients with Cardiomyopathy

**DOI:** 10.1155/2022/1390373

**Published:** 2022-09-01

**Authors:** Rongjin Huang, Feng Chen, Aiying Zeng, Jun Ke, Shirong Lin

**Affiliations:** ^1^Shengli Clinical Medical College of Fujian Medical University, Fuzhou 350001, China; ^2^Department of Emergency, Fujian Provincial Hospital, Fuzhou 350001, China; ^3^Fujian Key Laboratory of Emergency Medicine, Fujian Provincial Hospital, Fuzhou 350001, China; ^4^Fujian Provincial Institute of Emergency Medicine, Fuzhou 350001, China; ^5^Fujian Emergency Medical Center, Fuzhou 350001, China; ^6^Clinical Skills Teaching Center of Fujian Medical University, Fuzhou 350001, China

## Abstract

**Background:**

To determine the clinical significance of variations in serum sestrin2 protein levels in the development of septic cardiomyopathy in septic shock patients.

**Methods:**

The serum sestrin2 concentrations of each sample were determined using ELISA in a total of 67 control persons and 188 patients with septic shock. Furthermore, using transthoracic echocardiography, septic shock patients were split into two groups based on whether or not cardiomyopathy had developed, and the differences in each index between the two groups were analyzed. We looked at the relationship between serum sestrin2 levels, norepinephrine dosage, and NTproBNP levels. The influencing variables for the prediction of septic cardiomyopathy linked with the development of septic cardiomyopathy and clinical prognosis in septic cardiomyopathy were determined using multivariate binary logistic regression.

**Results:**

Assessment of left ventricular systolic function by measurement of LVEF revealed that 61/188 (32.4%) of the 188 patients with septic shock included in the research satisfied the diagnostic criteria for septic cardiomyopathy. (1) Sestrin2 protein levels showed a significant difference between septic shock and healthy controls (*p* < 0.01). (2) Compared to the group without septic cardiomyopathy, the group with combined septic cardiomyopathy had lower serum sestrin2 protein levels (*p* < 0.05), lower systolic blood pressure (*p* < 0.05), and higher plasma NTproBNP levels (*p* < 0.01) and used greater norepinephrine dosages (*p* < 0.01). The levels of serum sestrin2 protein revealed a little negative relationship with NTproBNP and norepinephrine dose. However, a binary logistic regression analysis revealed that none of these factors was an independent predictor of septic shock. (3) Age, lactate level, SOFA score, positive bacteremia, and sestrin2 protein were shown to be substantial discrepancies in clinical outcomes in patients with septic cardiomyopathy, becoming variables that impact clinical outcomes. Positive bacteremia (*p* = 0.031, OR = 5.084), SOFA score (*p* = 0.021, OR = 1.304), and sestrin2 protein (*p* =  0.039, OR = 0.897) were revealed to have independent influences in predicting clinical mortality outcome in septic cardiomyopathy using multivariate binary logistic regression.

**Conclusion:**

High serum sestrin2 levels clearly distinguish septic shock patients from healthy controls, whereas low serum sestrin2 levels are related with cardiac dysfunction to some extent but are not an independent influence factor for septic cardiomyopathy. Low serum sestrin2 levels were shown to be useful in predicting clinical outcome in patients with septic cardiomyopathy.

## 1. Introduction

Sepsis is a clinical condition in which the organism's inflammatory response to infection is disrupted, leading to physiological, biological, and biochemical problems. Sepsis and the release of pro-inflammatory mediators exceed the local environment, resulting in a broader reaction, multiple organ dysfunction syndrome (MODS), and mortality. The definition of sepsis is presently depicted as life-threatening organ dysfunction due to a dysregulated host response to infection, according to the 2016 Society of Critical Care Medicine/European Society for Critical Care Medicine (SCCM/ESICM) working group guideline criteria (sepsis-3) [1]. The Sequential (Sepsis-related) Organ Failure Assessment score [SOFA] corresponds to conscious function, pulmonary function, renal function, hepatic function, coagulation dysfunction, and other organ dysfunction parameters. Because the concept of distinguishing between septic myocardial depression, which represents a protective hibernation-type mechanism, and septic myocardial dysfunction, which represents a poor prognosis requiring effective treatment strategies, remains unresolved, there are only criteria for septic shock in the circulatory system and no criteria for septic cardiomyopathy. Although the exact definition and clinical significance of septic cardiomyopathy are unknown, studies have shown that the prevalence of septic cardiomyopathy in patients with septic shock ranges from 18% to 40%, and that the presence of septic cardiomyopathy increases mortality in patients with septic shock by 70% to 90% [2, 3], making early detection of septic cardiomyopathy and subsequent interventions critical.

We need to find effective identified biomarkers and potential novel therapeutic targets to optimize septic cardiomyopathy interventions and improve clinical outcomes. Compelling evidence suggests that the sestrin2 gene plays an important role in the maintenance of essential cardiac functions [4], and subsequent results from several team experiments have also shown that sestrin2 plays a protective role in a variety of cardiac diseases caused by stress [5]. Recent data have revealed that cardiac mitochondrial oxidative stress causes poor energy metabolism, which is important in the pathogenesis of septic cardiomyopathy [6]. Targeting and modulating the signaling route mediated by reactive oxygen species is a novel application, and oxidative stress-induced sestrin2 may be a crucial molecule in this system. Hwang et al. [7] showed that sestrin2 knockdown decreased AMPK phosphorylation in an in vitro model of cardiac H9C2 cell line and an in vivo model in C57BL/6 mice, resulting in downregulation of antioxidant enzyme expression, including catalase and superoxide dismutase, leading to increased ROS production in LPS-treated septic cardiomyopathy models and LPS-mediated myocardial fibrosis factors (e.g., type I and type III collagen) expression. Clinical data is based on the current belief that septic cardiomyopathy is a possible complication of septic shock [2], and we discovered that a previous clinical observational experiment conducted by Kim's research team found upregulated levels of sestrin2 protein expression in serum mononuclear cells in eight patients with septic shock compared to healthy controls [8]. However, because the study's clinical sample size was small, changes in sestrin2 in the serum of septic patients recovering from septic shock or exacerbation of septic cardiomyopathy, for example, were not tracked. There is no direct clinical trial data on the relationship between serum sestrin2 protein expression levels and septic cardiomyopathy. The goal of this study was to look at changes in serum sestrin2 protein levels in patients with septic shock using a large sample size, as well as to look into the clinical importance of serum sestrin2 protein in the development of septic cardiomyopathy in septic shock patients.

## 2. Materials and Methods

### 2.1. Study Subjects

**Figure 1:**
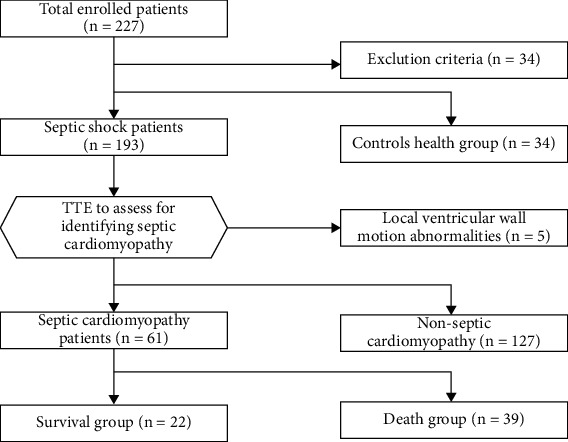
Study flowcharts for clinical research.

Patients with septic shock admitted to all intensive care unit (ICU) wards between September 2020 and March 2022 were recruited according to a protocol approved by the Fujian Provincial Hospital's Ethical Management Committee (NO.K2020-08-013), as well as healthy individuals screened by the Hospital Health Management Center during the same time period, and written informed consent was obtained from all recruits or their next of kin, as required by the Declaration of Helsinki. Septic shock was diagnosed according to the 2016 American Society of Critical Care Medicine/European Society for Critical Care Medicine (SCCM/ESICM) working group recommendations (sepsis-3) [1]. Retrospective cohort study trial design was used in this investigation. 227 patients with septic shock in all ICUs during the study period were observed to establish a retrospective cohort according to inclusion/exclusion criteria. The following were excluded [9–12]: (1) patient admitted to hospital with a history of coronary artery disease (CAD) and ECG suggestive of acute coronary syndrome, including ST-segment elevation or ST-segment depression and T-wave inversion (*n* = 12), and history of hypertension (*n* = 8), since plasma sestrin2 levels in patients with CAD and hypertension were reported to be high; (2) patients with diabetes mellitus (*n* = 6), because serum sestrin2 levels in patients with type 2 diabetes were reported to be high; (3) patients with oncology and immunodeficiency diseases (*n* = 5); (4) hospitalization <24 hours (*n* = 3). Thirty-four patients were excluded after applying inclusion and exclusion criteria, while five patients were also excluded after echocardiography was performed and found to have local ventricular wall motion abnormalities, and finally 188 patients with septic shock were included in this study; 67 healthy individuals screened by the health management center during the same period were selected (Figure 1).

### 2.2. Study Methods

#### 2.2.1. Echocardiography

The reversible nature of the course of septic cardiomyopathy may represent a protective hibernation-type mechanism, thus the clinical need to first identify patients in need of intervention [2], so reasonable criteria for enrolling cases in our clinical trial were as follows: (1) meeting diagnostic criteria for septic shock [1]; (2) patient having persistent hypoperfusion no matter adequate fluid resuscitation [13]; and (3) further transthoracic echocardiography (TTE) to assess decreased left ventricular systolic function as a criterion for identifying septic cardiomyopathy. According to our calculations, septic cardiomyopathy is often diagnosed by echocardiography 24 hours after admission, and within 72 hours. The most frequent method for determining left ventricular systolic function is to use the left ventricular ejection fraction (LVEF). The criteria are based on the American Society of Echocardiography's (ASE) guidelines for ventricular chamber measurement, which were updated in 2015. LV volumes were measured using a two-dimensional ultrasound tracing biplane disc method (modified Simpson method). And the following LVEF percentage ranges were incorporated: mild hypofunction (LVEF 40%-49%), moderate hypofunction (LVEF 30%-39%), and severe hypofunction (LVEF less than 30%) [14]. In addition, to improve the accuracy of the diagnosis, echocardiographic evaluation of LVEF to determine the index of left ventricular systolic function as a sign of septic cardiomyopathy requires additional information: (1) the left ventricular cavity size; (2) the severity of septic shock and the type and dose of catecholamines; (3) the exclusion of local ventricular wall motion abnormalities, dilated cardiomyopathy, hypertrophic obstructive cardiomyopathy, or the diagnosis of heart valve disease; and (4) the echocardiogram was repeated after 1 to 2 weeks of stable circulation. Previous and subsequent routine TTEs were done on the same ultrasonographer with substantial expertise in clinical patient unawareness, utilizing a GE-Vivid cardiac ultrasound system (USA) with an s3-Rs probe at 1.7-3.4 mHz.

#### 2.2.2. Blood Specimen Collection

Experienced nurses took blood samples from septic shock patients within 24 hours of admission and from healthy controls during a physical examination. Blood samples were obtained from research participants in procoagulant tubes, and the supernatant was centrifuged after 20 minutes at 1000 rpm as the sample for testing [15]. Serum samples were kept at a temperature of minus 80 degrees Celsius (blood collection to serum storage time not exceeding 45 minutes, which can be stored for 2 months for backup) for later use.

#### 2.2.3. Determination of Serum Sestrin2

An indirect double antibody sandwich technique enzyme-linked immunosorbent test was used to measure the serum sestrin2 concentration (ELISA). Sestrin2 protein assay kit source: Human ELISA Kit for sestrin2, Wuhan Yunclone Technology Co., Ltd, China, No. SEC840Hu. Performance parameters of the kit: detection range of 0.156-10 ng/mL, sensitivity, i.e., minimum detectable value of about 0.058 ng/mL, average intra-batch coefficient of variation of samples <10%, average inter-batch coefficient of variation <12%, recovery range of 92%-102%, average recovery 95%. Before use, all reagents and serum standards were gently brought to room temperature (18-25 degrees Celsius), and the following method was meticulously followed: For each test, standard curves were created, 7 standard wells and 1 blank well were set for various concentrations of standards in the kit, and 40 *μ*L of serum samples was added to 160 *μ*L of PBS (0.01 mol/L, pH = 7.0-7.2). Then, in turn, add 100 *μ*L of PBS, standard, and serum samples and incubate at 37 degrees Celsius for 1 hour; shake dry and add 100 *μ*L of assay solution A and incubate at 37 degrees Celsius for 1 hour; wash the plate three times and add 100 *μ*L of assay solution B and incubate at 37 degrees Celsius for 30 minutes; wash the plate five times and add 90 *μ*L of TMB substrate and incubate at 37 degrees Celsius for 10-20 minutes; add 50 *μ*L of stop solution to each well. Measure the optical density value (OD value) of each well by running the microplate reader and conducting measurement at 450 nm immediately. Create a standard curve with sestrin2 concentration on the *y*-axis and absorbance on the *x*-axis. The concentration of sestrin2 in the samples is then determined by comparing the O.D. of the samples to the standard curve.

#### 2.2.4. Demographic and Disease Data of Patients

Age, sex, previous illness, place, and kind of infection were all obtained as clinical baseline information for all research participants. At admission, ECG, blood pressure levels, and dosage of vasopressor norepinephrine were measured, as well as the 24-hour Acute Physiology and Chronic Health Evaluation (APACHE) II score and the Sequential (Sepsis-Associated) Organ Failure Assessment (SOFA) score. Routine blood test results, blood inflammation index PCT, blood biochemistry test results, lactate, cTNI (troponin I) levels, and N-terminal pro-brain natriuretic peptide (NTproBNP) levels were all taken. Patients provided bacterial culture findings and medication prescriptions. Indicators of clinical outcome, such as death during ICU stay, were gathered.

### 2.3. Statistical Analysis

Quantitative data with a normal distribution were characterized by mean and standard deviation and compared using an independent sample *t*-test. Non-normally distributed quantitative data were summarized as medians and interquartile ranges and compared using nonparametric tests. Proportions were used to statistically describe qualitative data, and chi-square tests or Fisher's exact tests were used to make comparisons. The association between serum sestrin2 protein, norepinephrine dosage, and NTproBNP levels was determined using Kendall's tau correlation analysis. To uncover independent influencing factors of septic cardiomyopathy, variables with *p* values less than 0.05 on univariate analysis were incorporated into a binary logistic regression analysis. Multicollinearity tests were carried out and evaluation metrics were expressed as dominance ratio (OR). For statistical analysis and graphing, GraphPad Prism 9.0 was used. A two-sided test with a *p* value less than 0.05 was judged significant.

## 3. Results

### 3.1. TTE for Diagnosis of Cardiac Function in Patients with Septic Shock

Among the 188 patients with septic shock included in the study, assessment of left ventricular systolic function by measurement of LVEF showed that a total of 61/188 (32.4%) patients met the diagnostic criteria for septic cardiomyopathy, including 45/61 (73.8%) with mild left ventricular hypoperfucton, 14/61 (23.0%) with moderate left ventricular hypoperfucton, and 2/61 (3.2%) with severe left ventricular hypoperfucton. After 1 to 2 weeks of circulatory stabilization, the results of echocardiogram in the surviving patients indicated that 29/29 patients (100%) had regained left ventricular function to greater than 50%. But the E/e′ value >14 or interval e′ velocity < 7 cm/s measured by tissue Doppler echocardiography during the recovery period as a criterion for left ventricular diastolic hypoperfucton revealed 11/29 patients (37.9%) with combined left ventricular diastolic hypoperfucton in the context of hemodynamic stability [2]. The findings supported our decision to use left ventricular systolic function as a foundation for categorizing septic cardiomyopathies that require therapeutic intervention, even if this might lead to underdiagnosis in some septic cardiomyopathy patients.

### 3.2. Comparison of Demographic and Disease Data among Combined Septic Cardiomyopathy Group, Non-Septic Cardiomyopathy Group, and Healthy Controls Group (Table 1 and Figure 2)

First, we discovered that even at an older baseline age of septic shock relative to healthy controls, sestrin2 protein levels strongly distinguished septic shock from healthy controls, despite earlier research showing that sestrin2 levels declined with advancing age [16, 17]. In addition, the group with combined septic cardiomyopathy had lower serum sestrin2 protein levels, lower systolic blood pressure, and higher plasma NTproBNP levels, as well as using larger norepinephrine doses than the group without septic cardiomyopathy. Baseline characteristics and clinical parameters such as age, gender, APACHEII score, SOFA score, hemoglobin, albumin, WBC count, PCT, lactate, and cTnI level (*p* > 0.05) showed no significant differences between the two groups.

### 3.3. Correlation of Serum Sestrin2 Protein Levels with Norepinephrine Dose and NTproBNP Levels (Table 2 and Figure 3)

The connection of sestrin2 levels with norepinephrine dosage and NTproBNP levels was determined using Kendallta correlation analysis. As indicated in Table 2, serum sestrin2 protein levels had a modest negative association with NTproBNP (*k* = −0.111, *p* = 0.024) and norepinephrine dosage (*k* = −0.120, *p* = 0.018).

### 3.4. Influence Factors for the Prediction of Septic Shock Associated with the Development of Septic Cardiomyopathy (Table 3)

The parameters linked to the prediction of septic shock associated with the development of septic cardiomyopathy were discovered using binary logistic regression analysis. The dependent variable was the occurrence or absence of septic cardiomyopathy. The variables with *p* values less than 0.05 on univariate analysis were then entered into a binary logistic regression analysis to further identify the independent predictor of septic cardiomyopathy; the study binary logistic regression analysis included systolic blood pressure, norepinephrine dose, NTproBNP levels, and serum sestrin2 protein. The result showed that none of these variables was the independent predictor of septic shock associated with the development of septic cardiomyopathy, corrected for age, sex, and SOFA.

### 3.5. Comparison of Clinical Parameters between the Survival and Death Groups in Patients with Septic Shock and Sepsis-Induced Cardiomyopathy (Table 4 and Figure 4)

Patients with septic shock were split into two groups based on their clinical outcomes: survival and death. The differences in each experimental index as well as the demographic characteristics of the two groups were explored. Age, lactate level, SOFA score, sestrin2 protein, and septic shock patients with sepsis-induced cardiomyopathy were shown to be significant disparities, becoming the factors that influence clinical outcomes in patients with septic shock. Furthermore, patients with septic cardiomyopathy were split into two groups based on their clinical outcomes: survival and death. The differences in each experimental index as well as the demographic characteristics of the two groups were explored. Age, lactate level, SOFA score, positive bacteremia, and sestrin2 protein were shown to be significant disparities, becoming the factors that influence clinical outcomes in patients with septic cardiomyopathy.

### 3.6. Logistic Regression Analysis of Factors Influencing Clinical Outcome in Septic Cardiomyopathy (Table 5 and Figure 5)

The parameters linked to clinical mortality outcomes in septic cardiomyopathy were investigated using binary logistic regression analysis. To explore binary logistic regression analysis, the survival and death groups of septic cardiomyopathy were used as dependent factors, while age, positive bacteremia, SOFA score, lactate, and serum sestrin2 protein levels were utilized as independent variables. Positive bacteremia (*p* = 0.031, OR = 5.084), SOFA score (*p* = 0.021, OR = 1.304), and sestrin2 protein (*p* = 0.039, OR = 0.897) were found to be independent impacts in predicting clinical mortality outcome in septic cardiomyopathy, according to the findings.

## 4. Discussion

### 4.1. How to Define Septic Cardiomyopathy

Based on the findings that normal myocardial lactate levels in blood collected from the coronary sinus of patients with cardiac dysfunction occurring in septic shock exclude myocardial ischemia and hypoxia [18], and the rare cell death detected by light microscopy, electron microscopy, and immunohistochemical staining for markers of cellular injury or stress in patients who died from sepsis-induced cardiac and renal dysfunction [19], as well as septic cardiomyopathy whose contractile function can return to normal when recovered from disease [20], we qualified septic cardiomyopathy as patients with abnormal cardiac pump function. Invasive hemodynamic monitoring is commonly used to detect aberrant cardiac pump performance in sepsis, but its drawbacks include the invasive procedures, as well as the complexity and uncertainty of the results' interpretation. Following the use of noninvasive imaging-based echocardiography, it has become a cornerstone in the identification of septic cardiomyopathy in patients with sepsis in the critical care unit. Although echocardiographic techniques have improved in recent years, they are still limited by standard differences and unknown clinical significance [2], so in this study, echocardiographic measurement of left ventricular ejection fraction was used to assess left ventricular systolic function in order to define septic cardiac dysfunction. A total of 61/188 (32.4%) patients satisfied the diagnostic criteria for septic cardiomyopathy in our study. After 1 to 2 weeks of stable circulation, echocardiographic results were repeated in 29/29 patients (100%) who had regained more than 50% of left ventricular function, while 11/29 patients (37.9%) had coupled left ventricular diastolic dysfunction in the setting of hemodynamic stability.

### 4.2. Interpreting the Study Results

We included a large sample size of clinical data of patients with septic shock by comparing plausible definitions of septic cardiomyopathy and choosing case studies with clinically important hemodynamic effects. The study's findings revealed that (1) serum sestrin2 levels were significantly elevated in patients with septic shock, clearly distinguishing septic shock from healthy controls; (2) serum sestrin2 expression in patients with combined septic cardiomyopathy was lower than in the group without septic cardiac dysfunction, and serum sestrin2 modest negative association with NTproBNP and norepinephrine dose, but none of these variables was an independent variable associated with the development of septic cardiomyopathy; (3) low serum sestrin2 level was an independent influence factor for predicting clinical mortality outcome in septic cardiomyopathy, according to the findings.

Our large sample size investigation found significantly higher serum sestrin2 levels in septic shock patients, which is similar with Kim et al.'s findings from a previous small sample clinical observation experiment [8]. The distinction is that we looked at circulating serum sestrin2 levels, whereas monocyte sestrin2 expression is what the latter looked at. In vivo, a range of tissue types, primarily macrophages, T lymphocytes, endothelial cells, cardiomyocytes, and cardiac fibroblasts, have been identified to express and release serum sestrin2 [21, 22]. However, due to the difficulty to perform myocardial pathological biopsies to assess myocardial tissue sestrin2 levels in clinical patients, our study was unable to give any information on the major source of circulating serum sestrin2 in patients with septic cardiomyopathy.

Increased levels of the circulating serum oxidative stress-inducible protein sestrin2 in patients with septic shock could be a compensatory response to increased oxidative stress aimed at preventing the progression of septic shock, whereas suppression of endogenous expression occurs with disease progression, such as the development of septic cardiomyopathy. To test this hypothesis, we report for the first time that circulating serum sestrin2 expression is lower in patients with combined septic cardiomyopathy than in patients without septic cardiomyopathy, and that serum sestrin2 and NTproBNP levels have a modest negative association correlation, but none correlation with serum cTnI levels. Septic troponin release may be generated by cytoplasmic leakage from cardiomyocytes rather than cell death [23], and our data demonstrated no significant difference in cTnI expression levels between the groups with and without septic cardiomyopathy. NTproBNP levels are used as an early diagnostic for fluid loading status and myocardial inhibition in individuals with septic shock because of their hemodynamic instability [24]. In this investigation, we found significant differences in NTproBNP levels in the subgroups with and without septic cardiomyopathy by using echocardiographic assessment of left ventricular ejection fraction to quantify left ventricular systolic function to characterize septic cardiac dysfunction. Despite the fact that sestrin2 and NTproBNP had predictive value for septic cardiomyopathy, neither measure was an independent predictor. Septic cardiomyopathy is a complicated pathophysiological process that involves numerous components such as decreased myocardial blood flow, direct myocardial inhibition, mitochondrial dysfunction, and oxidative stress [25].

Finally, this study showed that the regression coefficient of sestrin2 is -0.109< 0, *p* < 0.05, indicating that sestrin2 has a negative effect on the clinical death outcome of septic cardiomyopathy, and its OR value is 0.897, indicating that the chance of septic cardiomyopathy death with a one-unit higher in the value of sestrin2 protein was 89.7% of that with a one-unit lower, suggesting that sestrin2 protein plays a key role in predicting clinical mortality outcome in septic cardiomyopathy.

### 4.3. Oxidative Stress-Inducible Protein Sestrin2 and the Pathophysiology of Septic Cardiomyopathy

What are the mechanisms by which serum sestrin2 levels rise in sepsis and suppression of endogenous sestrin2 synthesis enhances the development of septic shock to septic cardiomyopathy? The sestrin2 gene is chromosomally localized at the 1p35.3 locus and has the capacity to encode 480 amino acids, producing a protein of approximately 55-60 kDa in size, with expression localized in cytoplasmic lysates [26] and possible mitochondrial localization [27]. To promote sestrin2 protein production, oxidative stress in septic organisms relies primarily on two transcription factors, P53 (Protein 53) and nuclear factor erythroid2-related factor 2 (Nrf2) [28, 29]. In septic organisms, oxidative stress-induced sestrin2 protein activation exerts antioxidative stress effects via (1) direct interaction with ROS when X-ray crystallographic methods were used to determine human sestrin2 structure [30], (2) inhibition of nicotinamide adenine dinucleotide phosphate oxidase (NADPH) and thus ROS production [31], (3) inhibition of ROS production via nuclear factor E2-related factor 2 (Nrf2) signaling pathway indirectly regulates Trx and Prx expression [32]; and (4) upregulates mitochondrial autophagy to remove damaged mitochondria and ROS in an AMPK-dependent and non-AMPK-dependent way [33, 34]; ULK1 mediates autophagy in the mitotic form of mitochondria [35]. In sepsis, endogenous targeting eventually restores mitochondrial function and protects cardiovascular function, whereas its gene loss or downregulation increases illness progression.

### 4.4. Limitations

The following are some of the study's limitations: (1) it is still a single-center, single-race study; (2) because myocardial biopsy was not possible, a correlation analysis between patient serum sesrin2 levels and myocardial tissue sestrin2 expression could not be performed, entailing future validation in clinical intervention experiments, in vitro cellular experiments, or in vivo animal experiments; (3) the study endpoints for clinical outcomes were only included in in-hospital mortality, necessitating future validation in clinical intervention experiments.

### 4.5. Conclusions

In conclusion, high serum sestrin2 levels clearly distinguish septic shock patients from healthy controls, whereas low serum sestrin2 levels are related with cardiac dysfunction to some extent but are not an independent influence factor for septic cardiomyopathy. Low serum sestrin2 levels were shown to be useful in predicting clinical outcome in patients with septic cardiomyopathy.

## Figures and Tables

**Figure 2:**
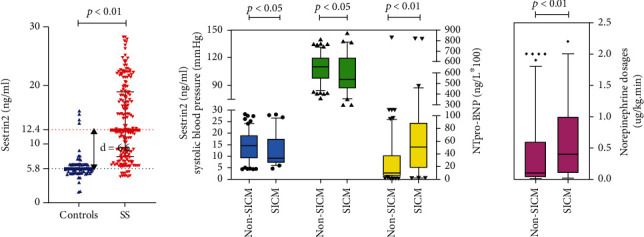
Comparison of demographic and disease data among combined septic cardiomyopathy group, non-septic cardiomyopathy group, and healthy controls group. Note. Controls: healthy controls group; SS: septic shock patients; no-SICM: non-septic cardiomyopathy group; SCIM: septic cardiomyopathy group.

**Figure 3:**
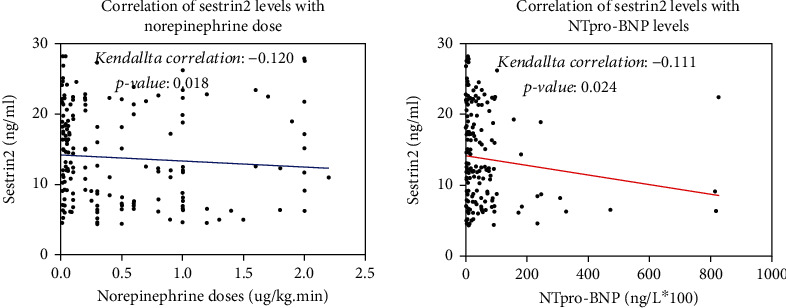
Correlation of serum sestrin2 protein levels with norepinephrine dose and NTproBNP levels.

**Figure 4:**
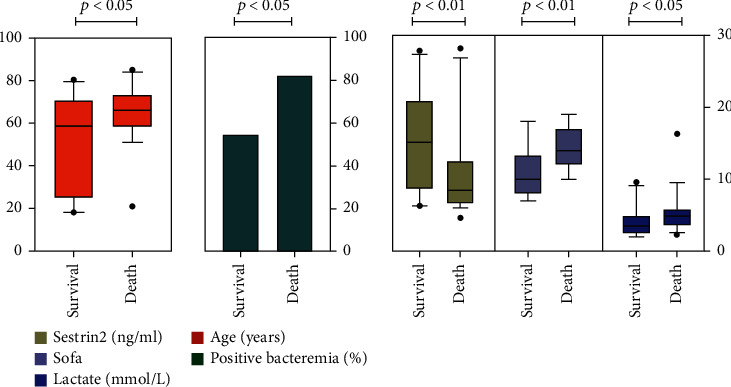
Comparison of clinical parameters between the survival and death groups in patients with septic cardiomyopathy. Note. Survival: survival groups in patients with septic cardiomyopathy; death: death groups in patients with septic cardiomyopathy.

**Figure 5:**
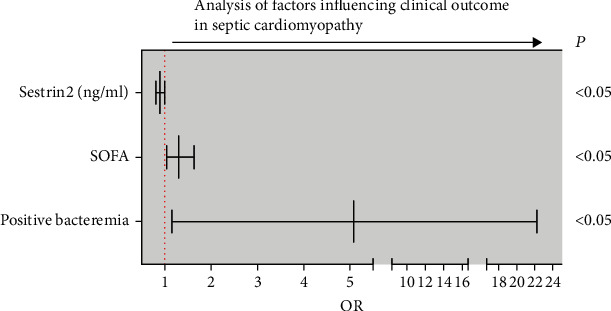
Logistic regression analysis of independent factors influencing clinical outcome in septic cardiomyopathy.

**Table 1:** Comparison of demographic and disease data among combined septic cardiomyopathy group, non-septic cardiomyopathy group, and healthy controls group.

	Control (*n* = 67)	*p*-value	SS (*n* = 188)	SS patients (*n* = 188)		
				Non-SICM (*n* = 127)	SICM (*n* = 61)	*p*-value
Age (years)	57 [46, 67]	< 0.01	65 [55, 72]	66 [55, 74]	65 [56, 72]	0.787
Sex (male%)	39 (58%)	0.670	115 (61%)	80 (63%)	35 (57%)	0.460
APACHEII	—	—	—	26 [19, 31]	26 [18, 32]	0.923
SOFA score	—	—	—	13 [10, 16]	13 [10, 17]	0.923
SBP (mmHg)	—	—	—	110 [97, 120]	96 [86, 120]	0.024^∗^
Hg (g/L)	—	—	—	101 ± 18	99 ± 20	0.627
Alb (g/L)	—	—	—	31 ± 6	30 ± 5	0.199
WBC count (×10^9^/L)	—	—	—	11.6 [7.6, 17.1]	14 [8.1, 18.6]	0.280
PCT (ng/L)	—	—	—	11.2 [2.6, 28]	19 [4.3, 41.5]	0.069
Norepinephrine (*μ*g/kg.min)	—	—	—	0.1 [0.03, 0.6]	0.4 [0.1, 1]	0.004^∗∗^
cTnI (mg/L)	—	—	—	0.03 [0.01, 0.24]	0.06 [0.02, 0.51]	0.071
NTproBNP (ng/L)	—	—	—	982 [524, 3727]	5040 [1765, 8920.5]	< 0.01^∗∗^
Sestrin2 (ng/mL)	5.8 [5.1, 6.6]	< 0.01	12.4 [7.9, 18.9]	14.6 [9.1, 19.2]	9.1 [7.3, 17.6]	0.043^∗^

Note. Controls: healthy controls group; SS: septic shock patients; no-SICM: non-septic cardiomyopathy group; SCIM: septic cardiomyopathy group; SBP: systolic blood pressure; Hg: hemoglobin; Alb: albumin.

**Table 2:** Correlation of serum sestrin2 protein levels with norepinephrine dose and NTproBNP levels.

	SBP	Norepinephrine	NTproBNP
Sestrin2	*K* *= 0.095*	*K* *= -0.120*	*K* *= -0.111*
	*p* *= 0.057*	*p* *= 0.018*	*p* *= 0.024*

**Table 3:** Influence factors for the prediction of septic shock associated with the development of septic cardiomyopathy.

	*β*	SD	Wald	*p*-value	OR	95% CI
SBP (mmHg)	-0.022	0.01	4.423	0.035	0.978	0.958-0.999
Norepinephrine (*μ*g/kg.min)	0.43	0.28	2.37	0.124	1.538	0.889-2.66
NTproBNP (ng/L)	0	0	7.201	0.007	1	1.000-1.000
Sestrin2 (ng/mL)	-0.026	0.027	0.959	0.328	0.974	.0.925-1.026
Constant	1.393	1.161	1.44	0.23	4.025	

CI: confidence interval; OR: odds ratio; SD: standard deviation; corrected for age, sex, and SOFA.

**Table 4:** Comparison of clinical parameters between the survival and death groups in patients with septic shock and sepsis-induced cardiomyopathy.

	SS		*p*-value	SICM		*p*-value
	Survival group (*n* = 128)	Death group (*n* = 60)		Survival group (*n* = 22)	Death group (*n* = 39)	
Age (year)	62 [50-72]	68 [62-74]	0.002^∗∗^	58 [25-70]	66 [58-73]	0.029^∗^
Sex (male%)	78 (60.9%)	37 (61.7%)	0.924	11 (50%)	24 (61%)	0.382
SOFA score	12 [10-16]	14 [12-17]	0.01^∗^	10 [8-14]	14 [12-17]	0.001^∗∗^
APACHEII score	25 [19-31]	26 [18-32]	0.799	25 [18-29]	26 [19-34]	0.685
PCT (ng/L)	11.7 [2.7-27.9]	18.2 [2.6-46.7]	0.459	20.3 [5.4-35.5]	19 [3.9-47.1]	0.839
Lactate (mmol/L)	2.6 [2.1-3.7]	4.1 [2.9-5.2]	<0.01^∗∗^	3.5 [2.6-4.9]	4.9 [3.6-5.8]	0.022^∗^
Positive bacteremia	86 (67.2%)	43 (71.7%)	0.537	12 (54%)	32 (82%)	0.021^∗^
Sestrin2 (ng/mL)	14.6[9.1-19.6]	9.0[6.8-17.9]	0.014^∗^	15.1[8.7-20.9]	8.5[6.7-12.5]	0.013^∗^
Infection site			0.360			0.442
Pulmonary	37 (28.9%)	19 (31.7%)		6 (27%)	15 (38%)	
Abdomen	56(43.8%)	30 (50%)		8 (36%)	17 (44%)	
Urinary tract	28 (21.9%)	6 (10%)		6 (27%)	4 (10%)	
Catheter elated	2(1.6%)	2 (3.3%)		1 (4%)	2 (5%)	
Others	5(3.9%)	3 (5.0%)		1 (5%)	1 (3%)	
SS with SICM	22 (17.2%)	39(65%)	<0.001^∗∗^			

Note. SS: septic shock patients; SICM: sepsis-induced cardiomyopathy.

**Table 5:** Logistic regression analysis of factors influencing clinical outcome in septic cardiomyopathy positive bacteremia.

	*β*	SD	Wald	*p*-value	OR	95% CI
Positive bacteremia	1.626	0.754	4.649	0.031	5.084	1.159-22.291
Age (year)	0.038	0.025	2.331	0.127	1.038	0.989–1.09
SOFA score	0.266	0.115	5.358	0.021	1.304	1.042–1.633
Lactate (mmol/L)	0.096	0.213	0.201	0.654	1.1	0.724–1.672
Sestrin2 (ng/mL)	-0.109	0.053	4.27	0.039	0.897	0.809–0.994
Constant	-5.22	2.056	6.444	0.011	0.005	

CI: confidence interval; OR: odds ratio; SD: standard deviation.

## Data Availability

Data are in supplementary information files.

## Supplementary Materials

Supplementary Materials The original clinical trial data, including clinical findings and observation outcomes, are contained in the supplemental material. This general report, which is derived from the patient's electronic case report format (eCRF), is used to rebuild and assess the clinical trial.
